# Burn scar pain: from mechanisms to treatments

**DOI:** 10.3389/fphys.2025.1627798

**Published:** 2025-09-24

**Authors:** Minjuan Zhao

**Affiliations:** Department of Burns, First People’s Hospital of Shangqiu City, Shangqiu, Henan, China

**Keywords:** burns, management, pain, scars (cicatrix), treatment

## Abstract

Chronic scars and pain following burns not only impair patients’ quality of life but also resist current empirical treatments, highlighting an urgent need for mechanism-based therapies. Early studies have characterized key mediators of scar fibrosis and nociception, yet integration of molecular and neural pathways remains limited. Here, we comprehensively review 1 molecular and cellular drivers of burn scar formation—particularly transforming growth factor-β (TGF-β)–induced fibroblast activation and extracellular matrix remodeling; 2 bidirectional interactions between scar tissue and nerve regeneration via neuropeptides (Nerve growth factor, Substance P, calcitonin gene-related peptide); 3 mechanisms underpinning long-term scar pain, including peripheral/central sensitization through TRPV1/Nav channels and neuroinflammation; and 4 emerging treatments—such as laser, extracorporeal shock wave therapy (ESWT), regenerative injections, and transient receptor potential (TRP) antagonists—that target these pathways. We conclude that a detailed understanding of scar–nerve crosstalk at the molecular level is pivotal for developing targeted interventions and improving long-term outcomes.

## 1 Introduction

Burn scars are the fibrotic tissue that forms during the healing of skin and subcutaneous injuries caused by thermal, chemical, electrical, or radiation exposure (Hettiaratchy and Dziewulski, 2004a). Depending on the healing trajectory, wounds may be classified as acute—characterized by timely epithelialization within a few weeks—or chronic, in which the repair process is prolonged (>12 weeks) and often results in excessive collagen deposition and structural remodeling (Peña and Martin, 2024). Such scars not only alter skin architecture but can also lead to functional impairment, cosmetic concerns, and persistent pain (Moi et al., 2016). The initial severity and depth of the burn injury critically influence both the likelihood and extent of scar formation and the persistence of pain symptoms. Clinically, burns are classified based on depth into superficial (involving only the epidermis), partial-thickness (superficial and deep dermal layers), full-thickness (extending through the entire dermis), and fourth-degree burns (involving underlying tissues such as muscle and bone) (Hettiaratchy and Dziewulski, 2004b; Jeschke et al., 2020). Deeper burns, especially full-thickness and fourth-degree injuries, are more prone to delayed healing, nerve damage, and consequent neuropathic pain, contractures, and long-term functional or cosmetic deficits (Jeschke et al., 2020).

Studies have estimated that 32%–72% of burn patients develop hypertrophic scars (Tyack et al., 2015), while the incidence of scar contractures at the time of discharge ranges from 38% to 54% (Téot et al., 2020). A substantial body of evidence indicates that survivors of extensive or deep burns often experience functional impairments due to scar contractures, reduced skin elasticity, and sensory abnormalities, which negatively impact daily activities and social participation (Téot et al., 2020). In addition to their disfiguring appearance, burn scars can cause symptoms such as pain, pruritus, and sleep disturbances, further diminishing quality of life. Chronic pain associated with burn scars persists in 25%–68% of patients and is frequently accompanied by neuropathic features such as tingling, burning sensations, and allodynia, posing significant challenges for clinical management (Bijlard et al., 2017). Neuropathological mechanisms including peripheral sensitization (Roy et al., 2023), neurogenic inflammation (Shahabi et al., 2009), and central sensitization (Stanton et al., 2024) are believed to underlie burn scar-related pain, with transient receptor potential vanilloid 1 (TRPV1), Nav channels, and neuropeptides playing pivotal roles in the transmission of pain signals.

Despite the availability of various analgesic and anti-scar interventions—such as pressure therapy, silicone dressings, laser treatments, and pharmacological approaches—the efficacy of these methods remains limited due to significant inter-individual variability and the lack of precise therapeutic targets. As a result, current strategies often fail to meet the diverse needs of burn patients. Therefore, elucidating the mechanisms underlying the interaction between scar formation and pain perception, identifying novel therapeutic targets, and developing multidimensional objective assessment tools are of critical importance for optimizing personalized treatment strategies (Faour et al., 2023). This review will explore the molecular and cellular mechanisms, neuropathological pathways, and the interplay between scar tissue and nerve regeneration. By integrating the latest research advances, we aim to identify potential translational points between mechanistic understanding and clinical application, thereby providing a theoretical foundation and research framework for future precision therapies. See Table 1 for a full list of abbreviations used.

**TABLE 1:** List of abbreviations.

Abbreviations	Full name
ADSCs	Adipose-Derived Stem Cells
CO_2_-AFL	Ablative Fractional CO_2_ Laser
AKT	Protein Kinase B
ATP	Adenosine Triphosphate
BDNF	Brain-Derived Neurotrophic Factor
BTX-A	Botulinum Toxin A
CBV	Cerebral Blood Volume
CCL2	C–C motif chemokine ligand 2
CGRP	Calcitonin Gene-Related Peptide
CNS	Central Nervous System
COL1A1	Collagen Type I Alpha 1
COL3A1	Collagen Type III Alpha 1
COX-2	Cyclooxygenase-2
DRG	Dorsal Root Ganglion
ECM	Extracellular Matrix
eNOS	Endothelial Nitric Oxide Synthase
Erbium-YAG	Erbium-doped Yttrium Aluminium Garnet Laser
ERK	Extracellular signal-Regulated Kinase
ESWT	Extracorporeal Shock Wave Therapy
LE-ESWT	Low-Energy Extracorporeal Shockwave Therapy
FAK	Focal Adhesion Kinase
FGF	Fibroblast Growth Factor
HGF	Hepatocyte Growth Factor
HRV	Heart Rate Variability
IL-10	Interleukin 10
IL-1β	Interleukin-1β
IL-6	Interleukin 6
iNOS	Inducible Nitric Oxide Synthase
JNK	c-Jun N-terminal kinase
LEP	Laser-Evoked Potentials
LLLT	Low-Level Laser Therapy
LOX	Lysyl Oxidase
M1/M2	Macrophage Phenotypes 1 and 2
MAPK	Mitogen-Activated Protein Kinase
miR-124	microRNA-124
miR-29	microRNA-29
MMP	Matrix Metalloproteinase
MRI	Magnetic Resonance Imaging
MSCs	Mesenchymal Stem Cells
Nav	Voltage-Gated Sodium Channel
NF-κB	Nuclear Factor-kappa B
NGF	Nerve Growth Factor
NK1	Neurokinin 1
nNOS	Neuronal Nitric Oxide Synthase
NRS	Numeric Rating Scale
p75NTR	p75 Neurotrophin Receptor
PGE_2_	Prostaglandin E2
Piezo1	Piezo-type mechanosensitive ion channel component 1
PKC	Protein Kinase C
p-mTOR	Phosphorylated Mammalian Target of Rapamycin
POSAS	Patient and Observer Scar Assessment Scale
PRGF	Plasma Rich in Growth Factors
PRP	Platelet-Rich Plasma
QST	Quantitative Sensory Testing
RCT	Randomized Controlled Trial
sCD163	Soluble CD163
sICAM-1	Soluble Intercellular Adhesion Molecule-1
SMAD	Small Mothers Against Decapentaplegic
SNAP-25	Synaptosomal-Associated Protein of 25 kDa
SP	Substance P
STAT3	Signal Transducer and Activator of Transcription 3
STM	Soft-Tissue Mobilization
sTREM-1	Soluble Triggering Receptor Expressed on Myeloid Cells-1
YAP/TAZ	Yes-associated protein and transcriptional coactivator with PDZ-binding motif
tDCS	Transcranial Direct Current Stimulation
TEAD	TEA domain family member
TEWL	Transepidermal Water Loss
TGF-β1/2/3	Transforming Growth Factor Beta 1/2/3
TNF-α	Tumor Necrosis Factor-α
TrkA	Tropomyosin receptor kinase A
TRP	Transient Receptor Potential
TRPA1	Transient Receptor Potential Ankyrin 1
TRPV1	Transient Receptor Potential Vanilloid 1
TSP-1	Thrombospondin-1
UNC 4P	University of North Carolina 4P Scar Scale
VAS	Visual Analog Scale
VEGF	Vascular Endothelial Growth Factor
VSS	Vancouver Scar Scale
wIRA	Water-Filtered Infrared-A
α-SMA	α-Smooth Muscle Actin

**FIGURE 1:**
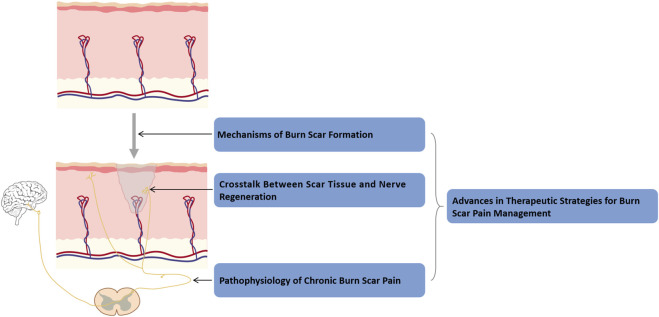
Roadmap of this review. This roadmap illustrates the organization of the review into four interconnected sections: mechanisms of burn scar formation, scar–nerve interactions, chronic scar pain pathophysiology, and advances in pain management strategies.

To guide readers through the organization of this review, we have provided a roadmap in Figure 1, which outlines the four main themes: Mechanisms of burn scar formation; Crosstalk between scar tissue and nerve regeneration; Pathophysiology of chronic burn scar pain; and Advances in therapeutic strategies for burn scar pain management. The following sections will address each of these topics in turn.

## 2 Molecular and cellular mechanisms of burn scar formation

**FIGURE 2:**
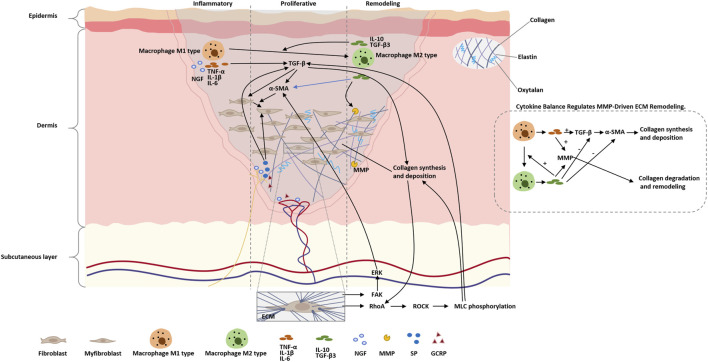
Schematic of molecular and cellular events in burn scar formation. The figure highlights the sequential phases (inflammation → proliferative → remodeling), key mediators (cytokines, growth factors, MMPs, neuropeptides), mechanotransduction pathways (Rho/ROCK, FAK/ERK) that drive myofibroblast activation, and the balance between pro- and anti-inflammatory signals which regulates MMP activity and final scar quality.

Burn scar formation is a highly dynamic and multi-layered process that begins with an acute inflammatory response at the site of injury, followed by a proliferative phase, and culminates in a remodeling phase where relatively stable scar tissue is formed (Werner and Grose, 2003). During this progression, fibroblasts transdifferentiate into myofibroblasts under the combined influence of transforming growth factor-β (TGF-β) signaling and mechanical tension (Tomasek et al., 2002), producing excessive type I and III collagen that contributes to the development of a fibrotic extracellular matrix (Wynn, 2008). Nerve growth factor (NGF) and neuropeptides such as Substance P (SP) and calcitonin gene-related peptide (CGRP) not only facilitate nerve and vascular remodeling but also modulate pain sensitivity within scar regions (Ji et al., 2014). The balance between early pro-inflammatory cytokines (e.g., TNF-α, IL-1β, IL-6) and later anti-inflammatory mediators (e.g., IL-10, TGF-β3) plays a critical role in regulating the activity of matrix metalloproteinases (MMPs), which in turn governs collagen degradation and remodeling efficiency—ultimately influencing the texture and functional quality of the resulting scar tissue (Page-McCaw et al., 2007). Figure 2 illustrates the main cellular and molecular mechanisms of the burn scar formation process. Table 2 summarizes the major cellular and molecular pathways involved in burn scar formation and pain modulation.

**TABLE 2:** Summary of major biological mechanisms and key signaling pathways in scar formation.

Topic	Key findings	Pathway/Mechanism	References
Fibroblast Activation and Collagen Deposition	Early α-SMA^+^ myofibroblast emergence TGF-β1/2–driven type I/III collagen deposition TGF-β3–mediated antifibrotic effect MMP-9/2–regulated ECM remodeling Mechanical tension enhances myofibroblast contractility	TGF-β1/2 → SMAD and non-SMAD TGF-β3 antifibrotic signaling MMP-9/2 → ECM turnover Rho/ROCK and FAK/ERK	Serini and Gabbiana (1996), Wang et al. (2011), Hinz et al. (2001), El Kahi et al. (2009), Ko et al. (2019), Walton et al. (2017), Chang et al. (2014), Cialdai et al. (2022), Nessler et al. (2014)
NGF and Neuropeptides	NGF from keratinocytes/macrophages/mast cells NGF–TrkA promotes sensory axon regeneration NGF upregulates VEGF/FGF and modulates MMPs SP via NK1/NF-κB drives fibrosis and pain	NGF → TrkA/p75^NTR VEGF/FGF induction MMP modulation SP → NK1 → NF-κB and TGF-β1 CGRP → TSP-1 and vasodilation	M et al. (2023), Di Sarno et al. (2024), Liu et al. (2021), Chung et al. (2020), Xing et al. (2024), Hochman et al. (2014), Lu et al. (2024)
Pro- vs. Anti-Inflammatory Balance	M1-derived TNF-α/IL-1β/IL-6 trigger fibroblast activation and pathological angiogenesis IL-6+VEGF sustain angiogenesis IL-10 via STAT3/AKT limits fibrosis and reprograms macrophages TGF-β3 suppresses collagen and promotes scarless healing	TNF-α/IL-1β/IL-6 signaling IL-6+VEGF → STAT3/AKT IL-10 → STAT3/AKT and M1→M2 and MMP regulation TGF-β3 regenerative signaling	Krzyszczyk et al. (2018), Johnson et al. (2020), King et al. (2014), Singampalli et al. (2020), Balaji et al. (2017), Larson et al. (2010), Rolfe and Grobbelaar (2012)

### 2.1 Fibroblast activation and collagen deposition

In burn scar formation, the transdifferentiation of fibroblasts into myofibroblasts—characterized by α-smooth muscle actin (α-SMA) expression—is a key initiating event for scar contraction and excessive tissue repair. Studies have shown that myofibroblasts emerge early during wound healing, with α-SMA-positive cells detectable as early as days 4–6 post-injury (Serini and Gabbiana, 1996). Their numbers increase significantly within 1–3 weeks, peaking around the second week, and while they gradually decline thereafter, they can persist in pathological scars (Wang et al., 2011). During this transdifferentiation, growth factors such as TGF-β1 and TGF-β2 activate both SMAD-dependent and non-SMAD signaling pathways to induce α-SMA expression in fibroblasts, endowing them with strong contractile capabilities and making them central players in scar contraction (Hinz et al., 2001). TGF-β1 is markedly upregulated in wound sites, promoting fibroblast proliferation, α-SMA expression, and the upregulation of pro-collagen and fibronectin genes via SMAD-dependent and independent mechanisms, thereby driving excessive type I and III collagen deposition (El Kahi et al., 2009). TGF-β2 is also elevated in the early repair phase, recruiting and activating fibroblasts, and enhancing the early secretion and crosslinking of type I/III collagen, which contributes to increased scar volume and stiffness (Ko et al., 2019). In contrast, TGF-β3 is highly expressed during early wound healing and has demonstrated antifibrotic properties across various tissues. It can inhibit collagen synthesis and promote scarless healing. For instance, Occleston et al. found that local delivery of TGF-β3 significantly reduced scar volume and suppressed type I/III collagen deposition, resulting in nearly scar-free tissue repair (Walton et al., 2017). Both animal models and clinical trials have shown that exogenous TGF-β3 can modulate the early inflammatory microenvironment and suppress fibroblast activation, offering new therapeutic insights into scar regression (Chang et al., 2014).

The remodeling of fibrotic matrix begins approximately 5–6 weeks post-injury and plays a pivotal role in determining the final texture and function of the scar (Cialdai et al., 2022). During this phase, MMPs, particularly MMP-2 and MMP-9, undergo activity changes that mediate partial degradation and reorganization of the extracellular matrix. MMP-9 levels increase rapidly within hours of injury, peaking on day 1 and gradually declining thereafter. MMP-2 levels rise between days 3–7, peaking around day 7 and remaining relatively stable to support sustained collagen degradation and reorganization (Nessler et al., 2014). In addition to biochemical signaling, mechanical tension serves as a critical trigger for fibroblast activation. *In vitro* and animal studies have demonstrated that external mechanical stretching can significantly enhance α-SMA expression and contractile force in myofibroblasts through Rho/ROCK and FAK/ERK pathways, exacerbating scar contraction and fibrotic matrix remodeling. Mechanical tension also promotes the proliferation of nerve fibers and the directional alignment of collagen in scar tissue, sensitizing nociceptive neurons and further amplifying scar-associated pain (Gallucci et al., 2006).

### 2.2 Nerve growth factor (NGF) and neuropeptides

During the early phase following burn injury, large amounts of NGF are secreted by various cell types, including keratinocytes, macrophages, and mast cells (M et al., 2023; Di Sarno et al., 2024). NGF signals through both the high-affinity receptor tropomyosin receptor kinase A (TrkA) and the low-affinity receptor p75 neurotrophin receptor (p75NTR), which together coordinate target cell proliferation, survival, and apoptosis (M et al., 2023). NGF-TrkA signaling stimulates the regeneration and collateral sprouting of sensory nerve axons post-injury, thereby accelerating the re-establishment of cutaneous neural networks and contributing to the restoration of sensory function (Liu et al., 2021). During the proliferative phase, NGF promotes angiogenesis by upregulating vascular endothelial growth factor (VEGF) and fibroblast growth factor (FGF) expression in endothelial cells and enhances re-epithelialization through increased proliferation and migration of epidermal stem cells (M et al., 2023).

Beyond its reparative functions, NGF in scar tissue has been shown to induce myofibroblast differentiation and modulate MMP activity, thereby attenuating excessive collagen deposition and scar contracture, leading to improved scar quality (Liu et al., 2021). Concurrently, neuropeptides released from C-fiber terminals—such as SP and CGRP—play dual roles in scar tissue by modulating nociception and pruritus, as well as altering the microenvironment through effects on vascular permeability and fibroblast proliferation (Chung et al., 2020). SP, a key mediator of neuropathic pain and post-injury itch, increases sensory nerve terminal sensitivity within scars, contributing to symptoms like burning, stinging, and hyperesthesia (Téot et al., 2020). It activates downstream inflammatory pathways (e.g., NF-κB) via the neurokinin 1 (NK1) receptor, upregulates profibrotic cytokines such as TGF-β1, and directly stimulates fibroblast proliferation and collagen synthesis, thereby promoting excessive scar tissue formation (Xing et al., 2024). CGRP, while also involved in pain transmission, predominantly modulates vasodilation and immune cell function (Téot et al., 2020). During the proliferative phase, CGRP enhances fibroblast proliferation and migration, and upregulates type I collagen synthesis, supporting granulation tissue maturation and wound closure (Xing et al., 2024; Hochman et al., 2014). Under certain conditions, CGRP exerts anti-inflammatory effects by inducing thrombospondin-1 release, suppressing excessive immune cell recruitment, and promoting immune cell apoptosis. Its potent vasodilatory capacity further improves local microcirculation and preserves capillary architecture (Xing et al., 2024; Lu et al., 2024).

### 2.3 Balance between pro- and anti-inflammatory mediators

In the early stages of wound healing, pro-inflammatory M1 macrophages release high levels of cytokines such as tumor necrosis factor-α (TNF-α), interleukin-1β (IL-1β), and interleukin-6 (IL-6), which promote fibroblast proliferation, angiogenesis, and keratinocyte migration, yet also lay the groundwork for excessive fibrosis (Krzyszczyk et al., 2018). These cytokines stimulate fibroblast proliferation and collagen secretion and act synergistically with angiogenic factors like VEGF to facilitate neovascularization. Moreover, they modulate basement membrane components and chemotactic signals, enhancing keratinocyte migration and accelerating re-epithelialization. IL-6, in cooperation with VEGF, sustains endothelial cell proliferation and migration, thereby maintaining pathological angiogenesis that supports prolonged fibroblast activity and continuous collagen deposition (Johnson et al., 2020).

As the wound enters the resolution phase, anti-inflammatory mediators such as IL-10 and TGF-β3 are upregulated, helping to suppress excessive inflammatory responses and facilitate extracellular matrix (ECM) remodeling (King et al., 2014). IL-10 activates downstream signal transducer and activator of transcription 3 (STAT3) and Protein Kinase B (AKT) pathways in fibroblasts, downregulating the expression of type I/III collagen and α-SMA, thus inhibiting the contractile phenotype of myofibroblasts and slowing the progression of fibrosis (Singampalli et al., 2020). IL-10 also reprograms macrophages from the M1 to the M2 phenotype, promoting immature vessel formation and regulating MMP activity to support organized ECM deposition and tissue repair (King et al., 2014; Balaji et al., 2017). In fetal wounds, TGF-β3 is more abundantly expressed compared to TGF-β1/β2. TGF-β3 suppresses collagen synthesis and modulates the inflammatory milieu, enabling regenerative, nearly scarless healing. As such, it is considered a key factor in scar reversal (Larson et al., 2010). Multiple animal studies have demonstrated that exogenous delivery of TGF-β3 significantly reduces scar formation and facilitates smooth, functionally superior tissue regeneration (Rolfe and Grobbelaar, 2012).

## 3 Interaction between scar tissue and nerve regeneration

During the later stages of burn wound healing, the mechanical rigidity of scar tissue and the biochemical remodeling of the ECM form a dual barrier to the regeneration of new nerve fibers. At the same time, neurotrophic factors and neuropeptides secreted by nerve axons activate fibroblasts in return, establishing a vicious feedback loop that collectively drives and maintains chronic neuropathic pain. Several studies have highlighted that mechanotransduction pathways, particularly Yes-associated protein and transcriptional coactivator with PDZ-binding motif (YAP/TAZ), act as key molecular integrators between ECM stiffness and fibroblast or neuronal responses (Dupont et al., 2011; Meng et al., 2018). The crosslinking of collagen fibers by lysyl oxidase (LOX), activation of integrins, and Hippo pathway inactivation lead to nuclear translocation of YAP/TAZ, which cooperatively regulate genes related to fibrosis and pain sensitization (Qin et al., 2018). Additionally, aligned ECM components like collagen I and laminin guide axons, whereas aberrant proteins such as Tenascin-C (Jiang et al., 2020; Guimarães et al., 2023), along with miRNA dysregulation (e.g., miR-29, miR-124), further disrupt regeneration and promote pain (van Rooij et al., 2008; Hassan et al., 2024; Shao et al., 2016).

### 3.1 Mechanical obstruction of scar matrix

LOX-mediated collagen crosslinking critically increases extracellular matrix stiffness and constitutes the principal mechanical barrier to nerve regeneration in burn scars (Tomasek et al., 2002). In burn scars, the activity of the LOX family of enzymes is significantly elevated, which promotes excessive crosslinking between type I/III collagen molecules. This results in an increase in the Young’s modulus of the scar tissue by 20%–50% compared to normal dermis, physically creating a “rigid cage” around the nerve growth cones. This increased rigidity significantly impedes the lateral extension of axons and induces persistent mechanical sensitization of local nerve endings (Cai et al., 2017; Chaudhari et al., 2023). The Hippo–YAP/TAZ pathway plays a central role in sensing this increased ECM stiffness. Under high mechanical stress, YAP and TAZ are dephosphorylated, translocate to the nucleus, and interact with TEA domain family member (TEAD) transcription factors to drive myofibroblast activation, α-SMA expression, and pro-fibrotic gene transcription (Dupont et al., 2011; Meng et al., 2018). YAP/TAZ also cooperate with TGF-β/SMAD signaling—through induction of SMAD7 and modulation of SMAD3 activity—to amplify extracellular matrix gene expression and reinforce tissue rigidity and reinforcing the mechanical barrier to nerve regeneration (Qin et al., 2018; Kuehlmann et al., 2020; Yan and Cui, 2023; Taskinen et al., 1995). Additionally, the mechanosensitive channel piezo-type mechanosensitive ion channel component 1 (Piezo1) is upregulated in both scar fibroblasts and sensory nerve terminals. Calcium influx mediated by Piezo1 activates the Rho/ROCK pathway, which not only promotes myofibroblast contraction and matrix remodeling but also induces repetitive firing in adjacent neurons, maintaining a state of mechanical hypersensitivity (He et al., 2024; Xu et al., 2023).

### 3.2 ECM’s role in nerve fiber guidance

In an ideal repair microenvironment, type I collagen and laminin guide axonal growth through integrin-ECM interactions, providing adhesion sites and directional guidance for the axons. Clinical studies have demonstrated that implantation of type I collagen conduits at facial nerve defects significantly enhances functional recovery, confirming the scaffold role of organized ECM in nerve regeneration (Yao et al., 2022). Decellularized fibroblast-derived matrices retain natural adhesion ligands and neurotrophic factor binding sites, significantly increasing the number and speed of axons crossing the injury gap in peripheral nerve injury animal models, further emphasizing the importance of biochemical scaffolds in promoting regeneration (Xu et al., 2023; Kim and Granstein, 2021). However, in pathological scars, the high expression of Tenascin-C and its multi-domain crosslinking networks at high concentrations misdirect nerve fibers, causing ectopic branching and disrupting nerve pathways within the fibrotic region. This misrouting leads to ectopic discharges, which become one of the sources of chronic pain (Jiang et al., 2020; Guimarães et al., 2023). The imbalance between mechanical and biochemical signaling is also amplified by the Piezo1 channel, whose calcium influx in response to mechanical stress in high-stiffness ECM disrupts biochemical signaling in nerve growth cones, triggering pathological pain responses (He et al., 2024; Xu et al., 2023). Furthermore, the imbalance in the expression of miR-29 family and miR-124 in fibrotic tissue and neurons not only regulates collagen synthesis genes but also influences the transcription of TRPV1 and Nav channels, molecularly cooperating to maintain scar-associated neuropathic pain (van Rooij et al., 2008; Hassan et al., 2024; Shao et al., 2016).

### 3.3 Nerve-mediated scar remodeling feedback

The abundant secretion of NGF from regenerating nerve terminals activates SMAD and mitogen-activated protein kinase (MAPK) signaling pathways in fibroblasts through the TrkA and p75NTR receptors. This significantly upregulates the expression of collagen type I alpha 1 (COL1A1), collagen type III alpha 1 (COL3A1), and α-SMA, promoting myofibroblast transdifferentiation and collagen deposition, which in turn increases scar stiffness and sustains a positive feedback loop of nerve sensitization (M et al., 2023; Micera et al., 2001). SP, released by C-fiber terminals and binding to the NK1 receptor, activates pro-inflammatory pathways such as nuclear factor-kappa B (NF-κB), enhancing neuronal excitability. It also directly promotes fibroblast proliferation and collagen synthesis, thereby maintaining a high activity pro-fibrotic and pro-nociceptive microenvironment at the scar edge (Zaarour et al., 2022). The continuous release of CGRP facilitates myofibroblast differentiation and matrix remodeling through both SMAD-dependent and -independent mechanisms. Its vasodilation and increased permeability effects further promote the infiltration of pro-inflammatory cells and nerve sensitization, strengthening the persistence of chronic pain under high-concentration conditions (Kim and Granstein, 2021). Additionally, pro-inflammatory macrophages in the ganglia and scar tissue interact through IL-1β/brain-derived neurotrophic factor (BDNF), activating microglial cells and sustaining p38 MAPK and epigenetic modifications in the central nervous system, thus constructing pain “memory” and consolidating long-term scar-related pain states (Guimarães et al., 2023; Zhang et al., 2023).

## 4 Pathophysiology of chronic burn scar pain

**FIGURE 3:**
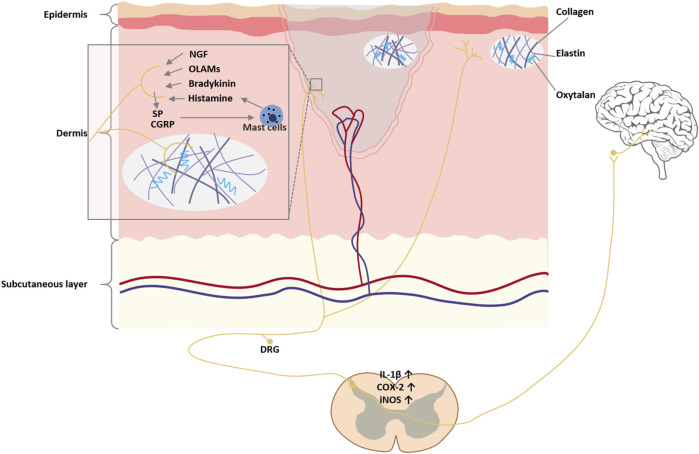
Pathways of burn scar pain. Aberrant nerve fiber regeneration within the scar, driven by elevated NGF, produces neuroma-like clusters and C-fiber hyperinnervation that lower pain thresholds and elicit spontaneous firing, while regenerated fibers tethered in rigid scar tissue generate traction neuropathy. Persistent peripheral nociceptor activation releases SP and CGRP, triggering mast cell degranulation and pro-inflammatory cytokine release, which in turn recruit immune cells and sensitize nociceptors, and sustained input activates spinal astrocytes and microglia to elevate IL-1β, cycloxygenase-2 (COX-2), and iNOS via NF-κB and c-Jun N-terminal kinase (JNK) pathways, producing central “wind-up”. Finally, excessive collagen deposition and cross-linking stiffen the scar—leading to mechanical stress, local ischemia, and microtrauma–inflammation feedback loops around joints or nerve pathways that perpetuate chronic pain.

Long-term pain arising from burn scars is multifactorial, encompassing peripheral nerve remodeling, ion channel sensitization, central neuroplastic changes, neuro-immune interactions, and the unique biomechanical properties of scar tissue. As summarized in Figure 3, burn scar pain arises from four converging mechanisms—aberrant nerve fiber regrowth and tethering within a rigid collagen matrix, neurogenic inflammation via SP/CGRP-driven mast cell activation, central sensitization through astrocyte/microglial release of IL-1β/COX-2/iNOS, and persistent biomechanical stress from stiff, inelastic scar tissue. The heterogeneity of pain phenotypes and enduring neural adaptations should be recognized to fully understand and manage chronic burn scar pain.

### 4.1 Peripheral nerve remodeling and pain phenotype heterogeneity

Burn scars often heal with dysregulated reinnervation, leading to neuroma‐like clusters and hyperinnervation that lower mechanical and thermal pain thresholds (Palanivelu et al., 2018). Elevated levels of NGF within the scar promote aberrant sprouting of C-fibers, exacerbating characteristic neuropathic sensations—burning, shooting, or electric shock–like pain—long after wound closure (Bijlard et al., 2017; Cuignet et al., 2015; Adenzato et al., 2018; Barrett et al., 2019). Moreover, regenerating axons may become entrapped within rigid scar matrices, creating traction neuropathy: scar-nerve adhesions restrict normal nerve gliding, so movement of surrounding tissues triggers ectopic discharges perceived as spontaneous or movement-evoked pain (Adenzato et al., 2018; Mewa Kinoo and Singh, 2017). Clinical observations reveal that burn scar pain is not monolithic. Patients exhibit diverse pain phenotypes—mechanical allodynia, spontaneous burning pain, and hyperalgesia—reflecting differential involvement of Aβ, Aδ, and C‐fiber subsets and their specific receptor expression profiles (TRPV1, TRPA1, Nav1.7) within the scar (Green et al., 2013; Gouin et al., 2017; Bagood and Isseroff, 2021). Psychological factors and individual coping strategies further shape these phenotypes, as pediatric survivors who rely on internalizing coping exhibit higher long‐term anxiety and pain levels (Griffin et al., 2015).

### 4.2 Ion channel sensitization and peripheral-central neuroplasticity

At the molecular level, burn‐induced pain involves upregulation and sensitization of key ion channels on primary afferents. TRPV1 channels are overexpressed and activated by lipid mediators in injured skin, and transient receptor potential ankyrin 1 (TRPA1) contributes to mechanical hypersensitivity; voltage‐gated sodium channels such as Nav1.7 are also overproduced, lowering activation thresholds and enabling spontaneous firing. Inflammatory mediators (e.g., bradykinin, TNF-α, IL-1β) amplify these effects via protein kinase C (PKC), NF-κB, and JNK pathways, perpetuating heightened nociceptor excitability (Green et al., 2013; Gouin et al., 2017; Bagood and Isseroff, 2021).

Continuous peripheral nociceptive input drives central sensitization. In rodent models, non-severe burns cause selective loss of large (Type A) dorsal root ganglia (DRG) neurons—with a relative increase in Type B (pain/itch) neurons—indicating lasting DRG remodeling that favors nociceptive signaling (Palanivelu et al., 2018). In the dorsal horn, sustained input activates microglia and astrocytes, elevating COX-2, iNOS, and pro-inflammatory cytokines, which further lower central pain thresholds and produce “wind-up” phenomena characteristic of chronic neuropathic pain (Lee et al., 2022; You et al., 2025).

### 4.3 Neuro-immune interactions and neurogenic inflammation

Sensory neurons in the scar release SP and CGRP, triggering mast cell degranulation and release of histamine, proteases, and cytokines. This neurogenic inflammation sensitizes nociceptors and recruits additional immune cells, creating a self-sustaining inflammatory loop at the scar site (Abd-Elsayed et al., 2022; Lee et al., 2022).

### 4.4 Scar biomechanics and sustained mechanical stress

**FIGURE 4:**
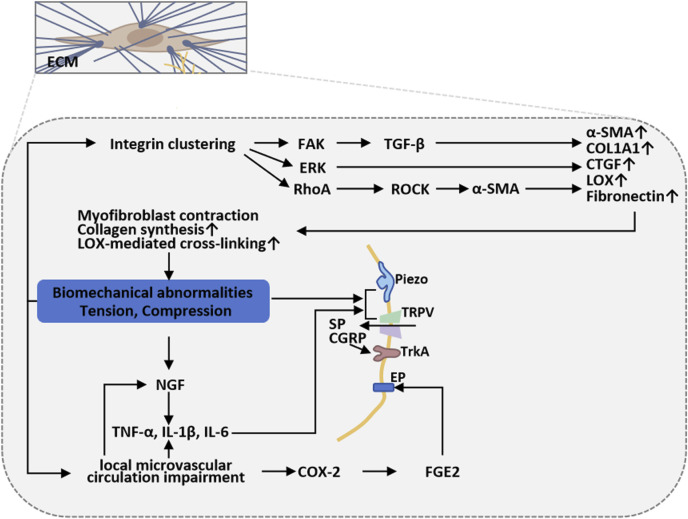
Scar biomechanics and sustained mechanical stress in burn-scar-related chronic pain. Myofibroblast-driven collagen cross-linking increases scar stiffness and adhesiveness, imposing sustained mechanical loads that activate nociceptors and mechanotransduction cascades (integrin/FAK, Rho/ROCK, FAK/ERK), reinforcing fibrosis. Concurrent activation of Piezo/TRP channels, abnormal nerve regeneration, neuroinflammation, and microvascular hypoxia sustains peripheral sensitization and drives central sensitization, maintaining chronic pain.

Biomechanical alterations of burn scars drive and sustain chronic pain through multilevel interactions (Morgan et al., 2018; Figure 4). In the course of burn wound remodeling, fibroblasts undergo phenotypic transition into myofibroblasts, leading to overproduction of densely cross-linked collagen. This excessive matrix cross-linking substantially augments scar stiffness and tissue adhesiveness, imposing continuous localized mechanical loads during skin traction or bodily movement, thereby activating peripheral nociceptors (Yin et al., 2022). The resultant mechanical milieu further drives fibrosis through canonical mechanotransduction cascades—integrin/focal adhesion-FAK, Rho/ROCK, and FAK/ERK—promoting sustained myofibroblast contractility and augmented matrix deposition. These processes reinforce each other to form a self-sustaining tension-fibrosis positive feedback loop (Yin et al., 2022). Furthermore, mechanical stimuli activate mechanosensitive ion channels—including the Piezo family and selected TRP channels—on peripheral sensory neurons and other cells, inducing neuronal depolarization and mechanical sensitization, which in turn amplify mechanical pain perception (He et al., 2021). Abnormal patterns of nerve regeneration are frequently observed within scar tissue, characterized by excessive proliferation and misdirected growth of nerve fibers, or entrapment within the fibrotic extracellular matrix, resulting in neuroma formation or small-fiber dysfunction. These alterations are accompanied by upregulation of neurotrophic factors such as NGF, SP, and CGRP, as well as neuroinflammatory mediators, which collectively exacerbate aberrant peripheral discharges and sustain neuropathic pain (Yang et al., 2025). In parallel, impaired microcirculation and focal hypoxia within the scar maintain a state of low-grade chronic inflammation. Repetitive and intense peripheral inputs may, via spinal and supraspinal pathways, induce or aggravate central sensitization, thereby establishing a chronic pain state driven by peripheral–central interplay (Yang et al., 2025).

Based on these mechanisms, clinical interventions that modify the mechanical environment (e.g., tension-reducing sutures, pressure therapy, silicone sheeting) or attenuate traction and neuronal excitability (e.g., local botulinum toxin injection, targeted inhibition of neurotrophic factors, or blockade of mechanosensitive channels) can partially alleviate scar-associated pain. These observations also highlight Piezo channels, the integrin–FAK/Rho signaling axis, and the NGF–neuroinflammation pathway as promising therapeutic targets for future intervention (Tiskratok et al., 2025; Kuehlmann et al., 2020).

## 5 Research progress of burn scar pain treatment

Burn-scar pain therapies have evolved from single‐modality palliation to multimodal, mechanism‐based regimens addressing fibrosis, aberrant innervation, and neuroinflammation. These approaches fall into six interrelated categories—laser therapies, ESWT, mechanical supports/topicals, manual and needling therapies, injection/regenerative medicine, and neuromodulation—converge on collagen remodeling, inflammation modulation, and nociceptor desensitization. To provide a clearer overview of current interventions targeting burn scar pain, Table 3 summarizes key treatment categories, specific interventions, mechanisms of action, clinical outcomes, and supporting evidence.

**TABLE 3:** Summary of burn scar pain treatments with mechanisms, outcomes, and evidence base.

Treatment category	Specific intervention	Mechanism of action	Key outcomes	Study design/Sample size	Reference
Laser Therapies	LLLT	Fractional photothermolysis → collagen remodeling	Significant improvement in VSS scores: treated areas decreased from 7.10 ± 2.13 to 4.68 ± 2.05; control areas decreased from 6.10 ± 2.86 to 5.88 ± 2.72	Prospective study; n = 19	Gaida et al. (2004)
	Fractional CO_2_ + Multiwavelength Lasers	Fractional photothermolysis and collagen remodeling improve pliability	Case report of late-stage napalm burns showed visible scar softening and improved texture	Case report/Case series; n = 1	Waibel et al. (2018)
	Fractional CO_2_ Laser (4 sessions)	Creates microthermal zones → collagen remodeling	VSS pliability ↓0.9 (2.29→1.39, P < 0.001); VSS vascularity and pigmentation improved; POSAS ↓ (P < 0.001)	Prospective study; n = 25	Khalid et al. (2025)
	Ablative fractional CO_2_ laser (CO_2_-AFL)	Fractional microthermal zones → collagen remodeling, ↓pliability	POSAS observer and patient scores improved; pliability and stiffness ↑ (P < 0.0001)	Prospective study; n = 49	Patel et al. (2019)
	Fractional CO_2_ Laser	Fractional photothermolysis → collagen remodeling	97.1% patients reported improvement in scar symptoms	Retrospective study; n = 170	Won et al. (2023)
	Multimodal Laser Therapy (CO_2_ + 1,540 nm + Dye Laser)	Fractional photothermolysis + non-ablative heating → collagen remodeling and scar contraction	scar pliability ↑ and volume ↓	Case report/Case series; n = 1	Campolmi et al. (2023)
	Fractional Erbium-YAG (2,940 nm)	Fractional photothermolysis → collagen remodeling	Significant improvement in UNC 4P Scar Scale scores (pain, pruritus, pliability, paresthesia); 94% patient satisfaction	Prospective study; n = 64	Madni et al. (2018)
	CO_2_-AFL	Fractional photothermolysis → collagen remodeling and scar contraction	Significant improvement in scar thickness, texture, color, and symptoms (pain and pruritus); quality of life increased by 15 points (median 120 to 135; p < 0.001)	Prospective study; n = 47	Issler-Fisher et al. (2017)
ESWT	ESWT	Mechanical stimulation → tissue regeneration	No significant improvement in scar appearance, pain, or pruritus compared to control group	RCT; n = 30	Aguilera-Sáez et al. (2022)
	Low-Energy Extracorporeal Shockwave Therapy (LE-ESWT)	Mechanical stimulation → tissue regeneration	pain ↓, pruritus ↓, health-related quality of life ↑	RCT; n = 45	Samhan and Abdelhalim (2019)

**TABLE 3:** () Summary of burn scar pain treatments with mechanisms, outcomes, and evidence base.

Treatment category	Specific intervention	Mechanism of action	Key outcomes	Study design/Sample size	Reference
	ESWT combined with Vivaphototherapy (wIRA)	Mechanical stimulation and phototherapy → improved blood perfusion, reduced inflammation, enhanced healing	Significant improvement in blood perfusion, inflammatory markers (CRP, IL-10, TNF-α, sICAM-1, sTREM-1, sCD163) ↓, better prognosis indicators	RCT; n = 120	Chen and Nie (2022)
	Low-energy ESWT (once weekly ×10, early remodeling)	Mechanical mechanotransduction → collagen remodeling, improved elasticity	Statistically significant improvement in skin elasticity (cutometry); no significant change in POSAS scores, redness, or transepidermal water loss (TEWL)	RCT; n = 40 (20 in ESWT, 20 plaebo)	Moortgat et al. (2020)
	ESWT on hand burn scars	Mechanical stimulation → reduced scar thickness, vascularity, pain, improved hand function	↓VAS pain (p = 0.001); ↓scar thickness (p = 0.018); ↓vascularity (p = 0.0015); ↑hand dexterity (card-turning p = 0.02; pegboard p = 0.004)	RCT; n = 48	Joo et al. (2020)
	Extracorporeal Shock Wave Therapy (100 impulses/cm^2^, 0.05–0.15 mJ/mm^2^, weekly ×3)	Mechanical stimulation → nociceptor inhibition, reduced fibrogenic signaling	Significant reductions in scar pain (NRS), increased pain thresholds, improved Nirschl scores and Roles and Maudsley ratings (all p-values <0.05)	RCT; n = 40	Cho et al. (2016)
Mechanical/Topical Agents	Silicone gel, pressure garment, or combined	Occlusion/hydration (→ collagen modulation); compression-induced apoptosis	Silicone alone group had slightly thinner scars vs. combined (mean diff = −0.04 cm; P = 0.05); no between-group differences in itch	RCT; n = 153	Wiseman et al. (2020)
	Monitored pressure garment (Smart suits)	Continuous compression → ↓collagen deposition, improved pliability	Early-start group (≤60 days post-burn) showed significant ↓ scar thickness, pigmentation, VSS pliability, pain, itch (all p < 0.01); late-start group had improvements except thickness and pigmentation	RCT; n = 34	Li et al. (2018)
	Topical ACD440 Gel (TRPV1 antagonist)	TRPV1 inhibition → reduced nociceptive signaling	Significant reduction in VAS pain and laser-evoked potentials (LEP); effect lasting at least 9 h	RCT; n = 24	Segerdahl et al. (2024)
	5% Lidocaine medicated plaster	Sodium-channel blockade → ↓sodium influx → ↓ectopic nociceptor firing	NRS pain ↓58.2% ± 27.8% (6.66→2.72); painful area ↓72.4% ± 24.7%; 69% functional improvement	Prospective study; n = 29	Correa-Illanes et al. (2010)

**TABLE 3:** () Summary of burn scar pain treatments with mechanisms, outcomes, and evidence base.

Treatment category	Specific intervention	Mechanism of action	Key outcomes	Study design/Sample size	Reference
Manual and Needling Therapies	Scrambler Therapy (MC5-A, 10 × 45 min sessions)	Non-invasive electrocutaneous stimulation → central pain network modulation	Significant VAS pain reduction vs. sham (p < 0.01); decreased cerebral blood volume in frontal and sensory cortices	RCT; n = 43	Lee et al. (2022)
	tDCS	Cathodal stimulation over sensory cortex → ↓ cortical excitability → ↓ pain anxiety	Significant reduction in pain anxiety scores (23.4 ± 3.8 vs. 29.3 ± 2.0, p ≤ 0.001); effect sustained post-stimulation	RCT; n = 30	Hosseini Amiri et al. (2016)
	Soft Tissue Mobilization	Manual manipulation improves tissue extensibility and remodeling of immature burn scars	Improved scar pliability and reduced scar thickness in immature scars	Pilot/Feasibility studies; n = 12	Silverberg et al. (1996)
	Burn Rehabilitation Massage	Mechanical stimulation enhances collagen remodeling, improves scar vascularity and elasticity	Hypertrophic scar thickness and redness ↓; improved scar pliability and symptoms	RCT; n = 30	Cho et al. (2014)
	Massage Therapy	Mechanical stimulation enhances tissue relaxation, reduces neural excitability, and improves local blood flow	Significant reduction in itching, pain, anxiety, and depression; improved mood	RCT; n = 20	Field et al. (2000)
	Depressomassage	Mechanical suction → skin fold mobilization → improved scar remodeling and barrier function	Minimal difference in scar color and TEWL between groups; no significant improvement with depressomassage	Pilot/Feasibility studies; n = 43	Anthonissen et al. (2018)
	Acupuncture	Local needling around scar tissue to modulate neuroinflammation and improve tissue remodeling	Short-term relief of pain and itch; significant reduction in scar thickness, redness, and pliability up to 6 months post-injury	Case report/Case series; n = 1	Tuckey et al. (2022)
Injection and Regenerative Medicine	PRP Injection	PRP contains growth factors that promote tissue healing and modulate inflammation, potentially alleviating neuropathic pain	Significant reduction in mechanical allodynia; decreased expression of inflammatory markers (e.g., p-mTOR, CCL2) in skin and spinal cord; improved tissue remodeling	Animal study/Preclinical; n = 6 per group	Huang et al. (2018)
	PRP and plasma rich in growth factors (PRGF)	Growth factors in PRP/PRGF promote wound healing and tissue regeneration	Reduced pain and inflammation	RCT; n = 60	García-Sánchez et al. (2022)
	Medical needling + Non-cultured Autologous Skin Cell Transplantation (ReNovaCell)	Needling stimulates collagen remodeling; cell transplantation aids repigmentation	Significant improvement in hypopigmented burn scars	Clinical study; n = 14	Busch et al. (2016)

**TABLE 3:** () Summary of burn scar pain treatments with mechanisms, outcomes, and evidence base.

Treatment category	Specific intervention	Mechanism of action	Key outcomes	Study design/Sample size	Reference
	Intralesional Botulinum Toxin Type A	Muscle relaxation and reduction of neurogenic inflammation	Significant reduction in post-burn pruritus compared to Triamcinolone	RCT	Hoseininejad et al. (2024)
	Intralesional Botulinum Toxin Type A	Reduces hypertrophic scar formation via decreased fibroblast activity	Decreased scar volume and itching	RCT	Tawfik and Ali (2023)
	Autologous fat grafting	Anti-inflammatory effect in scar tissue and spinal cord, modulating neuropathic pain pathways	Significant reduction in neuropathic pain and inflammation markers in scar and spinal cord	Animal study/Preclinical (rat model); n varies	Huang et al. (2015)
	Autologous fat grafting	Mechanical cushioning + immunomodulation reducing scar inflammation and nerve sensitization	Clinical improvement in neuropathic pain symptoms post severe burns	Case report/Case series	Fredman et al. (2016)
	Autologous ADSCs	Anti-inflammatory and neuroprotective effects reducing neuropathic pain	Reduced burn-induced neuropathic pain behaviors	Animal study/Preclinical (rat)	Lin et al. (2017)
Neuromodulation and Emerging Techniques	Auriculotherapy (magnetic beads on Shenmen and Subcortex)	Auricular stimulation → vagal activation, ↓pain and itch, improved sleep	↓VAS pain and itch; ↓5-D Pruritus scores; improved heart rate variability (HRV) parameters	Prospective study; n = 30	Chen et al. (2021)
	Electroacupuncture (3 × 30 min sessions)	Electrical needle stimulation → increases Aδ/C-fiber thresholds, reduces nociceptive hypersensitivity, modulating peripheral spinal circuits	VAS pain median decreased from 6.8 to 4.5 (p < 0.05); subgroup of responders (n = 18) showed normalization of Aδ/C thresholds; significant itch reduction in all patients	Prospective study; n = 32	Cuignet et al. (2015)
	Scrambler Therapy (10 × 45 min sessions over 2 weeks)	Patient-specific electrocutaneous stimulation → “scrambled” non-pain signals via C-fibers, modulating central pain network	Significant VAS pain reduction vs. sham (p < 0.01); post-treatment MRI showed ↓ cerebral blood volume (CBV) in orbitofrontal, middle and superior frontal gyrus and gyrus rectus; ↑ CBV in sensorimotor cortex on affected side	RCT; n = 43	Lee et al. (2022)

### 5.1 Laser therapies

Non‐pharmacologic modalities leveraging photobiomodulation and mechanotransduction have shown notable efficacy in controlled studies. Low‐level laser therapy (LLLT) at 400 mW, 670 nm decreased visual analog scale (VAS) pain scores and improved scar pliability for up to 3 months post‐treatment, mechanistically linked to mitochondrial chromophore absorption that enhances adenosine triphosphate (ATP) synthesis, modulates reactive oxygen species, and downregulates pro-inflammatory cytokines via NF-κB inhibition (Gaida et al., 2004; Mansouri et al., 2020). Waibel et al. reported a case of multi-wavelength fractional CO_2_ laser treatment performed over four decades after napalm burns, resulting in noticeable scar softening, improved texture, and better pliability (Waibel et al., 2018). While the report highlights the long-term potential of laser interventions, it remains a single-patient case study, underscoring the need for larger trials to confirm reproducibility. In a prospective cohort study involving 49 children undergoing 180 laser sessions, significant improvements were observed in both observer- and patient-rated patient and observer scar assessment Scale (POSAS) scores, with marked reductions in stiffness and improved pliability (Patel et al., 2019). Fractional CO_2_ laser produces microthermal treatment zones within hypertrophic burn scars, promoting controlled collagen remodeling and downregulating TGF-β1 and IL-6, which translates into significant reductions in scar thickness and a 97.1% patient-reported improvement rate in appearance, pliability, and pain (Won et al., 2023). A recent prospective study involving 25 patients with skin of color reported significant reductions in vancouver scar scale (VSS) pliability scores after four treatment sessions, along with improvements in pigmentation and patient-reported outcomes (Khalid et al., 2025). Its high reported patient satisfaction underscores its clinical promise, yet objective measures of pain and randomized control data are lacking. Larger-scale, blinded trials incorporating sensory profiling and neurophysiological correlates are needed to validate and refine these findings.

Multimodal regimens combining fractional CO_2_, non-ablative 1,540 nm, and 595 nm dye lasers synergistically soften scar bulk, target neovasculature, and modulate pro-inflammatory cytokines, sustaining comfort and reducing scar thickness for at least 3 months (Campolmi et al., 2023). The combination approach targets multiple scar features simultaneously, enhancing efficacy. Regimen complexity and cost may hinder widespread clinical adoption. Comparative studies evaluating cost-effectiveness, treatment sequencing, and long-term analgesic durability across different scar pain phenotypes are warranted. Adjunctive Erbium-YAG (2940 nm) and low-level soft-laser (670 nm) photobiomodulation further enhance collagen reorganization and mitochondrial function to alleviate neuropathic pain (Gaida et al., 2004; Madni et al., 2018; Issler-Fisher et al., 2017). These adjunctive modalities may optimize outcomes through synergistic mitochondrial and extracellular matrix effects. Nonetheless, their additive benefit over monotherapy remains insufficiently quantified.

### 5.2 Extracorporeal shock wave therapy (ESWT)

ESWT, reduced scar pain by overstimulating and “defunctionalizing” peripheral nociceptors, promoting fibroblast mechanotransduction through AKT signaling, and inducing hyaluronan-rich vesicle release that remodels extracellular matrix and decreases tissue stiffness (Cao et al., 2025). This mechanism-based approach directly targets mechanical contributors to neuropathic pain, offering an advantage in scars with high rigidity. But the underlying nociceptor subtypes affected remain poorly characterized. A single randomized trial suggests ESWT can improve scar appearance, pain, and pruritus over 5 months when added to standard care, but direct comparisons to standard care alone were not statistically significant (Aguilera-Sáez et al., 2022). Larger, longer-term randomized controlled trials (RCTs) with standardized dosing and patient stratification are needed to confirm and optimize ESWT’s clinical benefits in burn scar management. Repetitive micro-mechanical stresses activate fibroblast and endothelial mechanotransduction, upregulating endothelial nitric oxide synthase (eNOS)-mediated angiogenesis and downregulating SP, CGRP, and IL-6, which transiently desensitizes hyperexcitable nociceptors and diminishes neurogenic inflammation (Aguilera-Sáez et al., 2022; Samhan and Abdelhalim, 2019; Chen and Nie, 2022; Moortgat et al., 2020; Joo et al., 2020; Cho et al., 2016). The mechanotransduction effects provide a dual benefit for both scar remodeling and pain relief, supported by animal and human data. However, the analgesic duration is often transient, typically lasting several weeks. Longitudinal trials with extended follow-up are needed to assess sustained desensitization and recurrence risk.

Meta-analyses confirm significant VAS pain reductions (SMD = −0.59; p < 0.0001) and itch relief (SMD = −0.94; p = 0.004) versus standard care (Samhan and Abdelhalim, 2019). This quantitative synthesis strengthens the clinical relevance of ESWT. Heterogeneity across protocols (e.g., intensity, frequency, anatomical site) limits interpretability. Standardized treatment regimens and responder subgroup analyses should be integrated into future RCTs. Combining ESWT with water‐filtered infrared-A (wIRA) photobiomodulation further augments IL-10 and suppresses TNF-α and prostaglandin E2 (PGE_2_) to optimize the healing milieu (Chen and Nie, 2022). This synergistic protocol expands therapeutic impact to both inflammatory and regenerative pathways. Yet, controlled head-to-head trials comparing ESWT alone vs. ESWT+wIRA are lacking. Site-specific protocols for hand scars report superior functional outcomes and scar pliability over sham (Joo et al., 2020), though longer follow-up RCTs highlight the need to refine dosing parameters (Aguilera-Sáez et al., 2022). Targeted anatomical protocols allow for precision medicine applications and better functional recovery. However, reproducibility across different patient populations remains uncertain. More robust multicenter trials are needed to optimize dose, site, and patient-specific customization.

### 5.3 Mechanical support and topical agents

Silicone gel forms an occlusive, hydrating film that normalizes transepidermal water loss, preventing keratinocyte-driven overproduction of collagen via cytokine-mediated keratinocyte–fibroblast signaling. Pressure garments (15–25 mmHg) apply uniform compression, inducing localized hypoxia that promotes fibroblast apoptosis and realigns collagen fibers along normal skin tension lines, yielding modest reductions in scar height (−0.04 cm; P = 0.05) but inconsistent analgesic benefit (Wiseman et al., 2020; Harris et al., 2024; Li et al., 2018). These results highlight the limited and inconsistent analgesic and anti-hypertrophic effects of current mechanical approaches. While widely prescribed and integrated into burn rehabilitation protocols, clinical studies have reported variable adherence and limited sustained pain relief. Pressure therapy’s analgesic mechanisms remain poorly understood, necessitating mechanistic trials exploring its interaction with local nociceptor function and inflammatory modulation.

Recent efforts to modulate TRP channels have produced compelling early‐phase data supporting their role in scar‐associated nociception. Topical application of the selective TRPV1 antagonist ACD-440 gel resulted in significant reductions in visual analogue scale (VAS) scores and pinprick pain responses over 4 weeks in a Phase 2a trial, with minimal systemic exposure, likely by inhibiting TRPV1-mediated cation influx and subsequent neurogenic inflammation (Segerdahl et al., 2024). This agent represents a novel, targeted approach to peripheral sensitization with a favorable safety profile. However, the small sample size and short trial duration limit extrapolation. Preclinical work on TRPA1 antagonists such as LY3526318 has further confirmed the potential of targeting multiple TRP family members to attenuate chronic pain, underscoring the value of ion-channel–targeted approaches for neuropathic components of burn pain (Green et al., 2016; Salas et al., 2017; Mellado Lagarde et al., 2024). These mechanistic insights open avenues for multi-target topical therapies. Translational gaps persist as human trials of TRPA1 antagonists remain scarce. Topical 5% lidocaine plasters inhibit voltage-gated sodium channels in peripheral nerve terminals, reducing numerical rating scale (NRS) pain scores by 58.2% ± 27.8% and decreasing painful surface area by 72.4% ± 24.7%, with 69% of patients reporting functional gains (Correa-Illanes et al., 2010). This modality offers rapid, localized analgesia with a favorable tolerability profile and minimal systemic side effects. However, long-term use may be limited by cost, adherence, and tolerance development. Comparative studies assessing lidocaine plasters versus ion-channel antagonists would help determine optimal first-line topical therapy.

### 5.4 Manual and needling therapies

Neuromodulation approaches targeting central pain networks have produced encouraging preliminary results. Scrambler therapy—using algorithmically varied electrocutaneous stimuli to “scramble” pain signals transmitted via C-fibers—yielded significant pain reductions in chronic burn patients over 2 weeks. Concurrent functional magnetic resonance imaging (MRI) revealed normalization of activity within the pain matrix, supporting its central neuromodulatory mechanism (Lee et al., 2022). This non-pharmacologic technique shows promise for central desensitization, but current evidence stems from small, uncontrolled cohorts. Transcranial direct current stimulation (tDCS) over the primary motor cortex decreased pain anxiety and improved pain thresholds in burn patients, likely through enhancement of descending inhibitory pathways and modulation of cortical excitability, although effects on itch and pain intensity warrant further study (Hosseini Amiri et al., 2016; Lefaucheur et al., 2017). The method is non-invasive and well-tolerated, but optimal stimulation parameters, duration of effect, and responder profiles remain to be defined. Mechanical therapies also play a role.

Mechanical manipulation disrupts fibrotic architecture and gates nociceptive signaling. Soft-tissue mobilization (STM) applies sustained shear and compression to break aberrant collagen cross-links, realign fibers, and enhance microvascular perfusion. Pilot RCTs note within-group ROM gains and subjective pain relief, albeit without significant between-group differences. While STM is safe and low-cost, its additive benefit in multimodal protocols is unclear and requires larger trials with objective outcome metrics (Silverberg et al., 1996). Scar massage (30 min twice weekly) employs effleurage and Petrissage to stimulate Aβ fibers, invoking spinal gate-control analgesia and achieving an additional 1.2-point VAS reduction versus controls (Cho et al., 2014; Field et al., 2000). Randomized trial (Anthonissen et al.) has rigorously evaluated a manual/needling modality—depressomassage—in addition to standard physiotherapy. Over 6 months, adding depressomassage did not improve scar colour, transepidermal water loss, or pain (VAS) compared with physiotherapy alone (Anthonissen et al., 2018). No controlled trials of soft-tissue mobilization, scar massage, acupuncture, or neuromodulatory techniques (e.g., scrambler therapy, tDCS) were identified, highlighting a critical need for well-designed RCTs to establish their efficacy in burn-scar pain management. Perilesional acupuncture combined with scar massage activates deqi-related central analgesic pathways, producing sustained NRS pain decreases for up to 6 months post-treatment (Tuckey et al., 2022). This integrative regimen demonstrates durability, but sample sizes were small and control groups varied. Overall, mechanical manipulation can plausibly disrupt fibrotic architecture and gate nociceptive signaling. However, high-quality, adequately powered RCTs with objective outcome measures (for example, blinded ROM assessment, validated pain scales, perfusion or ECM imaging, and MMP/TIMP biomarkers) are needed to define the magnitude, durability, and optimal role of these interventions in burn-scar pain management.

### 5.5 Injection and regenerative medicine

Bioactive injections target both scar tissue and nociceptive pathways. Bioactive injections target both scar tissue and nociceptive pathways. Rodent models show a 45% reduction in collagen deposition and increased mechanical withdrawal thresholds at weeks 7–8 (Huang et al., 2018), suggesting concurrent anti-fibrotic and analgesic effects. In a randomized intra-patient trial, platelet-rich plasma (PRP) accelerated donor-site healing (55% vs. 20% epithelialization by day 8, p = 0.036) and improved pain/scar outcomes versus hydrocolloid dressings (García-Sánchez et al., 2022). These findings underscore the dual benefits of injectable strategies in modulating both mechanical and sensory properties of scar tissue. The translation of these results to humans remains preliminary, highlighting the need for dose-escalation studies and long-term safety data. Separately, combining medical needling with non-cultured autologous skin cell suspension achieved significant repigmentation in 85% of participants at 12 months (Busch et al., 2016). Botulinum toxin A (BTX-A) cleaves synaptosomal-associated protein, 25 kDa (SNAP-25) to block acetylcholine and neuropeptide release, and inhibits fibroblast proliferation, producing greater scar-thickness and pruritus reductions than triamcinolone acetonide (P = 0.0287; P = 0.0482) (Hoseininejad et al., 2024; Tawfik and Ali, 2023). While these results mirror preclinical dual anti-fibrotic and analgesic effects, the field lacks randomized, placebo-controlled, long-term trials for agents like botulinum toxin A, adipose-derived stem cells, and cytokine modulators.

Anti-inflammatory modulation within the scar microenvironment has emerged as another promising avenue. Autologous fat grafting into burn scars, rich in adipose-derived stem cells, significantly downregulated pro-inflammatory cytokines (IL-1β, TNF-α) and enzymes (COX-2, iNOS, nNOS) within both scar tissue and spinal dorsal horns, correlating with reduced mechanical allodynia and thermal hyperalgesia in animal models (Huang et al., 2015; Fredman et al., 2016; Lin et al., 2017). The mechanism involves suppression of NF-κB and JNK signaling pathways, leading to decreased neuroinflammation and spinal neuronal apoptosis. This dual peripheral-central mechanism suggests that ADSC-based therapy may interrupt chronic pain circuits beyond the scar. There is a need for larger-scale, placebo-controlled human trials to evaluate durability and consistency of the effect. Building on these preclinical data, small open‐label trials of the IL-1 receptor antagonist anakinra in patients with chronic hypertrophic scars are underway, aiming to attenuate peripheral nociceptor sensitization and central neuroinflammatory cascades (Huang et al., 2018; Lin et al., 2017). The rationale is strong, given the role of IL-1β in both pain initiation and maintenance. However, human data are currently limited to early-phase exploratory studies.

Regenerative cell therapies seek to restore normal dermal architecture and nerve patterning while mitigating fibrosis. Mesenchymal stem cell (MSC)-based therapies aim to restore dermal structure, promote ordered nerve regeneration, and mitigate fibrosis. A Phase 1 dose-escalation study of intravenous mesenchymal stem cells (MSCs) in acute burn patients reported accelerated wound closure and reduced scar thickness. Secondary analyses noted lower patient-reported pain scores during rehabilitation, likely reflecting MSC-mediated paracrine release of anti-fibrotic (HGF, IL-10) and neurotrophic factors that promote orderly reinnervation (Lin et al., 2017). While this represents a promising avenue for mechanism-based repair, regulatory inconsistencies and cost barriers currently limit widespread implementation. Autologous fat grafting, through the adipose-derived stem cells (ADSCs), promotes angiogenesis, reduces inflammation, and regulates immune responses by releasing growth factors and cytokines, thereby alleviating scar-related pain. By reducing extracellular matrix stiffness and improving tissue compliance, it may reduce mechanical strain on regenerating nerve fibers—thereby attenuating pain (Ahmad et al., 2024). This mechanical-biological synergy is appealing in post-surgical scar management. However, variability in graft take and resorption rates poses a challenge to reproducibility. Optimization of delivery techniques and integration with other regenerative methods may enhance outcomes.

### 5.6 Neuromodulation and emerging techniques

Targeted electrical or magnetic stimuli directly modulate pain circuits. Auricular magnetic-bead stimulation of Shenmen and Subcortex enhances parasympathetic tone, reducing VAS pain from 4.8 ± 1.2 to 2.6 ± 1.0, though effects partially revert by 1 month (Chen et al., 2021). This technique is low-cost, non-invasive, and easily repeatable, offering a valuable option for patients with contraindications to systemic therapy. However, its effects appear transient, with partial symptom rebound within 1 month. Electroacupuncture elevates Aδ and C-fiber thresholds and yields significant pain-score reductions in responders (Cuignet et al., 2015). Its analgesic effects likely involve spinal segmental modulation and endorphin release, offering a dual peripheral–central mechanism.

Spinal cord stimulation offers opioid-sparing analgesia in refractory burn pain and permits permanent implantation with cessation of opioid use. Its long-term implantation capability and central targeting offer advantages in persistent neuropathic pain states. Its invasive nature, high cost, and risk of complications (e.g., infection, lead migration) limit its use to select, treatment-resistant patients. Scrambler therapy algorithmically “scrambles” C-fiber input to normalize aberrant pain signaling, demonstrating significant, durable VAS reductions and central network modulation on MRI (Lee et al., 2022). Its central desensitization mechanism is particularly relevant to chronic burn pain, which often involves spinal sensitization. Though promising, scrambler therapy currently lacks large-scale validation, and its optimal treatment schedule and durability beyond several months remain unclear.

### 5.7 Biomarker-driven stratification and personalized approaches

Burn scar pain represents a multifaceted clinical challenge, with patients exhibiting predominantly inflammatory-driven pain, peripheral neuropAthic pain, or centrally sensitized pain. Biomarker-driven stratification offers the potential to categorize patients based on dominant pain mechanisms, thereby guiding individualized, mechanism-specific interventions. For instance, patients exhibiting elevated levels of pro-inflammatory mediators such as IL-1β and TNF-α in scar tissue and plasma may represent an inflammatory-dominant phenotype. These patients could benefit preferentially from anti-inflammatory and immunomodulatory therapies, such as IL-1 receptor antagonists or adipose-derived stem cell (ADSC) grafting, which have been shown to downregulate inflammatory cytokines and alleviate neuroimmune sensitization both peripherally and within the spinal cord (Huang et al., 2015). This subset may also respond favorably to regenerative biologics with anti-fibrotic properties targeting NF-κB and JNK pathways (Fredman et al., 2016; Lin et al., 2017).

Upregulation of ion channels—particularly TRPV1 and TRPA1—has been observed in patients with peripheral sensitization phenotypes, marked by thermal hyperalgesia and mechanical allodynia. In such cases, TRPV1 antagonists like ACD-440 have demonstrated significant reductions in VAS pain scores in early-phase clinical trials, with favorable tolerability and minimal systemic exposure (Segerdahl et al., 2024). Targeting these ion channels with topical or injectable formulations could yield personalized relief in patients identified through molecular or sensory profiling (Segerdahl et al., 2024; Green et al., 2016; Salas et al., 2017).

For patients whose pain persists despite peripheral intervention—and in whom neuroimaging reveals sustained cortical hyperactivity or altered pain network connectivity—a centrally sensitized phenotype may be inferred. These individuals may benefit more from neuromodulatory interventions such as tDCS, Scrambler therapy, or spinal cord stimulation (Cuignet et al., 2015; Lee et al., 2022; Hosseini Amiri et al., 2016; Lefaucheur et al., 2017). Functional MRI and quantitative sensory testing (QST) can assist in identifying central amplification patterns, enabling early selection of central nervous system (CNS)-directed therapies.

Despite these advances, implementation remains limited by several barriers. Most biomarker studies to date are exploratory, underpowered, and lack standardization. Multicenter prospective trials with defined mechanistic endpoints are needed to validate stratification frameworks and assess predictive utility. Moreover, integration of these biomarkers into real-time clinical workflows will require user-friendly platforms, possibly augmented by artificial intelligence and digital health technologies.

Several critical gaps persist in burn‐scar pain research despite recent advances. Most clinical trials remain small and use heterogeneous outcome measures, limiting meta‐analytic synthesis and generalizability (Deflorin et al., 2020; Nguyen et al., 2025). There is no consensus on validated, burn‐specific pain assessment scales—particularly for pediatric and darker‐skinned populations—leading to underrepresentation and measurement bias (Nguyen et al., 2025; Jeschke et al., 2020). Long-term follow-up beyond 1 year is uncommon, hindering evaluation of durability and potential late‐emerging adverse effects of emerging therapies (Jeschke et al., 2020; Dobson et al., 2024). Correlative studies linking molecular or imaging biomarkers to clinical pain outcomes are scarce, obstructing biomarker-driven patient stratification and mechanism-based treatment optimization (Siu et al., 2025; Atiyeh et al., 2025). Head-to-head comparisons of TRP-channel antagonists, cell-based regeneration, photobiomodulation, and neuromodulation are lacking, precluding evidence-based selection among modalities (Siu et al., 2025; Amini-Nik et al., 2018). Regulatory and manufacturing inconsistencies in cell therapies impede reproducibility across centers and raise cost-effectiveness concerns that remain largely unaddressed (Siu et al., 2025), ENREF77 (Atiyeh et al., 2025). Moreover, the potential of digital health platforms and artificial intelligence for personalized pain management in burn scars remains unexplored, representing a promising yet untapped frontier.

## 6 Conclusion

Scar formation after burn injury is a multi-phase, multifactorial process regulated by complex interactions. The TGF-β signaling pathway plays a central role by promoting the transdifferentiation of fibroblasts into myofibroblasts and enhancing collagen I/III deposition, thereby contributing to the excessive fibrotic matrix. NGF and neuropeptides such as SP and CGRP not only facilitate peripheral nerve regeneration but also exert bidirectional regulatory effects within the scar microenvironment by modulating angiogenesis and fibroblast proliferation.

A dynamic balance between pro-inflammatory cytokines (e.g., IL-6, TNF-α, IL-1β) and anti-inflammatory cytokines (e.g., IL-10, TGF-β3) is maintained throughout the early, middle, and late stages of scar development, jointly influencing matrix remodeling and scar maturation. In peripheral sensitization, upregulation of TRPV1 and Nav channels leads to reduced pain thresholds, while neurogenic inflammation and central sensitization—mediated through neuropeptide release, microglial activation, and epigenetic modifications—sustain and amplify chronic pain signaling. The high stiffness and organized collagen structure of scar ECM act not only as mechanical barriers but also, under specific conditions, provide biochemical cues for nerve fiber guidance. The balance between these opposing properties is crucial for nerve regeneration and pain perception.

In parallel with these mechanistic insights, burn scar pain management has evolved toward multimodal, mechanism-based strategies. Contemporary interventions include laser therapy and ESWT to promote collagen remodeling and reduce scar stiffness, as well as adjunctive topical agents and pressure garments to modulate inflammation and attenuate hyperalgesia. Regenerative injection therapies (such as stem cell or platelet-rich plasma injections) aim to reverse fibrosis and foster tissue regeneration. Neuromodulation techniques and TRP-channel antagonists (for example, TRPV1 inhibitors) are being applied to desensitize nociceptors and modulate central pain networks. In addition, biomarker-driven stratification also offers new opportunities to tailor burn scar pain treatments based on individual pain mechanisms. These modalities collectively address the peripheral and central contributors to chronic scar pain. However, robust outcome measures and long-term efficacy for these treatments require further validation.
